# Non-targeted proteomics of acute respiratory distress syndrome: clinical and research applications

**DOI:** 10.1186/s12953-021-00174-y

**Published:** 2021-03-20

**Authors:** Xu-Peng Wen, Yue-Zhong Zhang, Qi-Quan Wan

**Affiliations:** 1 Transplantation Center, the Third Xiangya Hospital, Central South University, Changsha, 410013 Hunan China; 2 Clinical Medicine, Xiangya School of Medicine, Central South University, Changsha, 410083 Hunan China

**Keywords:** ARDS, Non-targeted proteomics, Clinical applications, Study designs

## Abstract

Acute respiratory distress syndrome (ARDS) is characterized by refractory hypoxemia caused by accumulation of pulmonary fluid with a high mortality rate, but the underlying mechanism is not yet fully understood, causing absent specific therapeutic drugs to treat with ARDS. In recent years, more and more studies have applied proteomics to ARDS. Non-targeted studies of proteomics in ARDS are just beginning and have the potential to identify novel drug targets and key pathways in this disease. This paper will provide a brief review of the recent advances in the application of non-targeted proteomics to ARDS.

## Introduction

Acute respiratory distress syndrome (ARDS) is a clinical syndrome caused by various pulmonary and extrapulmonary factors, characterized by refractory hypoxemia due to accumulation of pulmonary fluid [1]. There are more than 3 million patients with ARDS every year in the world. 10% of the patients in the intensive care unit (ICU) were admitted due to ARDS. The mortality rate of which was as high as 37.5% [2].

In recent years, with the development of molecular biology and bioinformatics, a variety of omics research methods have been applied to ARDS research, including genomics, transcriptomics, proteomics, and metabolomics [3], which have greatly accelerated the pace of ARDS research. Among them, as the main executor of life activities, proteomics plays an important role in the researches.

Proteome refers to all proteins expressed in a genome, a cell or tissue. This concept was first proposed by Marc Wilkins in 1994 [4]. Proteomics can capture a complete set of expressed proteins in an organism, including protein isoforms and post-transcriptional modifications. By identifying the differentially expressed proteins or the whole set of proteins expressed in tissues or blood samples, we can analyze and understand the protein changes in the process of disease, find out the key targets, and study the corresponding genes and metabolites, to provide a starting point for exploring the pathogenesis, early diagnosis and treatment of the disease.

Although the application of proteomic technology to the study of the pathogenesis of ARDS has just started, its great potentials in deepening the understanding of protein expression patterns, discovering new injury mediators, and developing new therapeutic drugs have emerged [5]. In this paper, we will review the proteomics of ARDS reported in recent years, which is summarized as follows.

## Acute respiratory distress syndrome (ARDS)

ARDS is a clinical syndrome characterized by high permeability pulmonary edema, which results in diffuse alveolar damage and often multiple organ failure [6, 7]. The mortality of ARDS has declined considerably due to the advance in mechanical ventilation settings but still stays as high as 35–46%. Such high mortality of ARDS patients means that no effective drug therapy is available for it yet. Here are several reasons for this phenomenon. First of all, ARDS is a comprehensive result of several pathways [8, 9], including endothelium injury and activation, epithelial injury, inflammation, coagulation, oxidative stress and metabolic dysfunction. Therefore, it is not effective enough to have treatment for a single protein or pathway. Secondly, since ARDS is a clinical syndrome resulting from different causes, such as pneumonia, bloodstream infections, lung contusion, shock and burn injury, are is divided into two aspects, direct and indirect causes [10], so it is difficult to cure ARDS targeting for one primary disease. Thirdly, The Berlin definition of ARDS addressed limitations of the American-European Consensus Conference definition, but poor reliability in applying some criteria by clinicians, and it was reported that such a degree is not useful to assess the severity and preview prognosis [2].

Some achievements have been made in the study of ARDS biomarkers, such as angiopoietin-2, surfactant proteins, glutathione, selectins, thrombomodulin, adenosine, Clara cell protein and many other biomarkers, which were reviewed before [8, 9, 11–14]. Whereas, several clinical trials failed since there were several pathways instead of a single one causing ARDS. Moreover, researchers majored in specified fields. Therefore, it means traditional methods to find biomarkers are too limited to be comprehensive. In consideration of such a phenomenon, non-targeted proteomic research was applied for ARDS.

## Non-targeted proteomics

It is well known that the function of a cell is mainly determined by proteins rather than genes. Between a gene and its corresponding protein, great changes exist including the tissue-specific expression of genes, post-transcriptional modifications, post-translational modifications, protein-protein interactions and self-regulation of protein abundance. Therefore, proteomics is more practical and challenging than genomics. Non-targeted proteomics is a powerful domain to discover and assess proteins unbiasedly and quantitatively or semi-quantitatively. Several reviews have introduced and summarized the detailed technologies and methods of non-targeted proteomics [11, 15–19], which would be omitted in this review.

### Development and comparisons of targeted versus non-targeted proteomics

Strategies for performing proteomic experiments are classified as either ‘targeted’ or ‘non-targeted. Targeted proteomics involves multiple analyses of known proteins and has been proved useful in assessing responses to the occurrence and progression of disease in the body. In contrast, non-targeted proteomics tries to detect as many different features as possible in a single analysis, and is combined with multivariate statistics to determine the biomarkers that distinguish cases and control groups. The application of the two groups of strategies is often confusing, so we summarize the advantages and disadvantages of the two groups of strategies in the following Table 1.

**Table 1:** Comparison of targeted and non-targeted proteomics

	Advantages	Disadvantages
**Non-targeted proteomics**	a. Broad spectrum screening of disease-related proteins b. Testing the samples and transitioning to targeted proteomics c. To detect as many distinct features as possible in a single analysis and, combined with multivariate statistics, identify biomarkers which distinguish case from control groups d. Allows larger scale studies of carefully phenotyped patients will identify novel pathophysiology in the disease	a. The target protein cannot be well screened for absolute quantitative and qualitative research and analysis b. Not entirely unbiased. The lack of absolute quantification hampers benchmarking of ‘normal’ protein levels and ultimately interlaboratory comparison of results c. False identification of proteins or bias/signal drift introduced from matrix effects may also occur due to a lack of standards
**Targeted proteomics**	a. To study different subtypes of the same disease type and draw a clinical reference map of biological targeted proteins, so as to guide clinical treatment b. Excellent analytical precision c. Unequivocal identification that can serve individually and together as biomarkers of both subphenotypes of disease and disease prognosis d. To reduce false positives which may lead to misinterpretation of an affected biological pathway	a. Limited coverage of the proteome which increases the risk of overlooking the proteomic response of interest. b. When a narrower mass range was applied, these proteins were not observable in samples. c. Limited resources and/or sample size and in the absence of prior knowledge on the protein of interest

In theory, the weaknesses encountered in one proteomic approach are the respective strengths of the other one. Although the disadvantages of non-targeted proteomics determine that we cannot screen target proteins for absolute quantitative and qualitative research and analysis, which may be caused by different types of diseases and multiple internal and external factors, it cannot deny the important role of non-targeted proteomics in disease analysis. Over the past decade, non-targeted proteomics has offered a growing potential to identify new biomarkers compared to targeted proteomics.

### Non-targeted quantitative proteomic technologies based on mass spectrometry

Since the concept of proteomics was put forward in 1994, quantitative proteomics has become the focus of proteomic research. It is to detect the difference in the quantity of total proteins expressed in normal and diseased tissues. Protein quantification technology in quantitative proteomics has also become an important way to discover biomarkers [20, 21]. In recent 10 years, with the rapid development of high-precision biological mass spectrometry (MS) and data processing technology, quantitative proteomic technologies have become the mainstream analysis method [22].

**Fig. 1:**
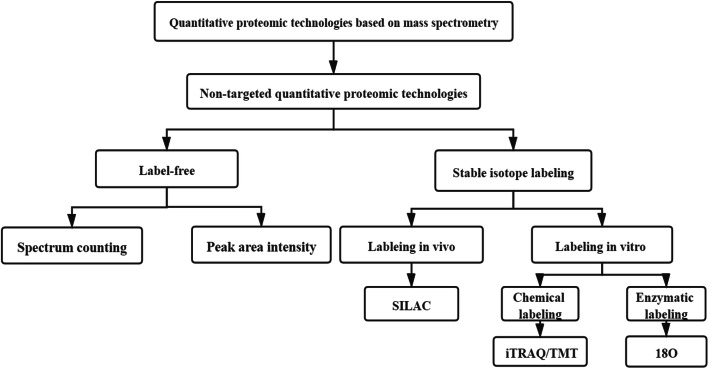
Classification diagram of non-targeted quantitative proteomic techniques. This figure depicts the workflow of non-targeted quantitative proteomic techniques. SILAC: Stable isotope labeling by amino acids in cell culture; iTRAQ: Isobaric tags for relative and absolute quantitation; TMT: Tandem mass tags

Non-targeted quantitative proteomic technology is for the undifferentiated analysis of all proteins in the samples, according to whether the proteins or peptides are labeled or not. It can be divided into label-free and stable isotop labeling. The advantages of this technique are low cost and simple sample preparation, and the quantitative error caused by sample pretreatment can be avoided by mixing different samples for mass spectrometry detection at the same time. Overall, the technical classification of non-targeted quantitative proteomics is shown in Fig. 1.

Notably, cell-free components, such as plasma and lung epithelial lining fluid have little DNA or RNA, but may have large numbers of proteins that are important markers of disease [16]. Up to now, proteomics has been widely used in studies of respiratory diseases, especially lung cancer, chronic obstructive pulmonary disease, asthma, pneumonia and idiopathic pulmonary fibrosis [11]. Whereas few pieces of research focused on ARDS [12]. On the basis of previous proteomic studies of ARDS, advances in non-targeted proteomic techniques and methodologies have made it possible to use bronchoalveolar lavage fluid (BALF), lung tissue, blood, and exhaled air condensate for pulmonary proteomic studies [16].

## Non-targeted proteomics of ARDS

From the first research of non-targeted proteomics in ARDS in 2004 [23], there were 16 studies in this field including 9 studies of human samples and 7 studies of rat or cell models. By analyzing the previous proteomic data, we can better understand the pathogenetic factors, signals, and events underlying ARDS. All these studies are summarized in Table 2.

**Table 2:** Some representative non-targeted proteomics studies in ARDS

Reference	Samples	Study objective	Number of subjects	Number of identified proteins;	Top 5 up/down regulated proteins	Proteomics methodology
[23] Bowler RP 2004	Plasma; Edema fluid in ARDS; BALF	To expatiate the protein profiles	16 ARDS; 12 healthy	300 distinct protein spots; 158 proteins identified	↑: albumin, IgG, transferrin, clusterin and hemoglobin α2; ↓: Surfactant protein A, α1-antitrypsin, haptoglobin, GST and transthyretin	2D-PAGE; MALDI-TOF/MS
[24] De Torre C 2006	BALF	To assess pulmonary inflammatory markers	11 ARDS; 33 healthy controls challenged with endotoxin	Only differentially expressed proteins reported	↑: Apo A1, S100A8, S100A9, AT III and transthyretin	SELDI-TOF/MS; 2D-PAGE; MALDI-TOF/MS
[25] Schnapp LM 2006	BALF	To collect a more complete protein profile	3 ARDS; 6 healthy	226, 291 and 659 proteins for the three patients studied	↑: albumin, ceruloplasmin, fibrinogen α chain, α1 chymotrypsin, α2-HS-glycoprotein and antitrypsin inhibitor	2D-HPLC; Shotgun Proteomics; LC-MS/MS
[26] Gessner C 2008	Exhaled breath	To find biomarkers of ARDS from EBC	24 ARDS; 10 healthy; 6 pneumonia (no ARDS)	3 proteins	↑: cytokeratins 2, 9, and 10	2D-PAGE; MALDI-TOF/MS
[27] Chang DW 2008	BALF	To examine changes of protein expression by time	ARDS on days 1 (*n* = 7), 3 (*n* = 8), and 7 (*n* = 5); 9 healthy	991 proteins spots seen. Only 80 proteins spots analyzed by MS which represented 37 unique proteins	↑: C3, S100A9, fibrinogen α chain, α1-antitrypsin, Apo A1 and hemopexin precursor	2D-PAGE; MALDI-TOF/MS
[28] Chen X 2013	Pooled plasma	To expound novel biomarkers and identify the potential ARDS treatment targets	11 ARDS (Direct lung injury = 6, Indirect lung injury = 5); 15 healthy	132 proteins	↑: SAA, isoform 1 of CRP, α1-antichymotrypsin, Leucine-rich α2-glycoprotein and α1-AGP1 ↓: complement factor H, Apo A1, serotransferrin, Apo C3 and Apo B100	MALDI TOF/TOF; iTRAQ; LC-MS/MS
[29] Nguyen EV 2013	BALF	To distinguish VAP in ARDS patients	30 ARDS: 14 VAP (+) and 16 VAP (−); 5 healthy	76 proteins	↑: in VAP (+): S100A8, elastase 2, lactotransferrin, actinin 1 and calnexin; In VAP (−): β-hemoglobin, keratin 2, fibrinogen beta chain, SERPINF1 and fibronectin 1	2D-HPLC; ESI-MS/MS
[30] Dong H 2013	Alveolar macrophage	Comparative analysis of alveolar macrophage proteome in patients with ARDS	14 ARDS (severe infection): 6 Severe pancreatitis, 4 acute pyogenic cholangitis, 4 acute ileus	27 proteins	↑: (at the recovery phase): GSTP1, PEX13, S100A8/A9 and leukocyte elastase inhibitor; (at the exudative phase): HSP27, annexin A8, cathepsin B, napsin A and galectin-3	2D-PAGE; MALDI-TOF/MS
[12] Bhargava M 2014	Pooled BALF	To differentiate the proteomic profiles in that survivors from non-survivors	7 early phase ARDS survivors; 8 Early phase ARDS non-survivors; 7 late phase ARDS survivors	724 proteins identified; 499 proteins quantified	↑: (in early survivors): AT III, coagulation factor II/XII, plasminogen, complement C5/C1r, and hemopexin (in early non-survivors): type I/III/V collagen and MMP 9	iTRAQ; 2D-HPLC; LC-MS/MS
[31] Liu D 2014	lung tissue	To obtain global protein expression changes in ARDS lung tissues and find new therapeutic target of ARDS	*Pseudomonas aeruginosa*-induced ALI rats	18 proteins	↑: peroxiredoxin 1, haptoglobin, GAPD, TCTP and VDBG; ↓: GSTA4, LGALS5, SPB1, transthyretin and Rho-associated protein kinase 1	MALDI-TOF/MS
[32] Tao J 2016	lung tissue	To determine the mechanism underlying JGT treatment of ARDS	3 groups (Con, Mod, and JGT-H)	67 proteins	↑: Actn2, Ligp1, Serpina3n, Mcm2, and Myl4; ↓: Hbbt1, Hbbt2, Coq9, Agrn and S1pr1	LC/MS; iTRAQ LC Triple-TOF
[33] Xu X 2017	lung tissue	To investigate the relationship between differentially expressed proteins (DEPs) and the pathogenesis of oleic acid (OA)-induced ALI in mice	OA-treated ALI rat model and saline-treated mice	849 proteins were differentially expressed between the two groups, including 545 upregulated and 304 downregulated proteins	↑: C1qA, ASCC3, Piwi-like protein 2, hemoglobin β and Cxcl4; ↓: Collagen Alpha-1(V) Chain, GNAI1, chorion protein S18, VAMP3 and C-C motif chemokine 21a	iTRAQ
[34] Tao Z 2017	macrophage cells and lung tissue	To explore the protective and therapeutic mechanisms of SFJDC in a rat model	LPS-induced ALI rat models and saline-treated mice	4 proteins in lung tissues;23 overlapping candidate proteins in AMs	↑: TNFAIP8, β-hexosaminidase submit α, 5-oxoprolinase, apoptosis-inducing factor 1 and histone-arginine methyltransferase CARM1 ↓: MAPRC1, DNAJB11, PRKCDBP, DNAJC5 and MAP6	HPLC-MS / MS,
[35] Ji Z 2017	serum of rat	To find proteins may be detected in the plasma of patients at high risk of ARDS	Rat model of ARDS was established by cecal ligation and puncture surgery	38 differentially expressed immunogenic proteins	↑: RASL11A, LOC689092, NUDT5, ENO1 and NEMF ↓: CHDH, Ankrd24, TEC, Phosphoglycerate kinase 1 and EPC1	2D-PAGE; MALDI-TOF/TOF
[14] Janga H 2018	H441 epithelial cells and endothelial cells	To analyse the site-specific effects of LPS on the ACB and reveal the effects on the individual cell types and the ACB as a functional unit	H441 epithelial cells and endothelial cells	5 proteins	↑: ICAM-1, VCAM-1, Angiopoietin 2, Macrophage colony-stimulating factor 1, complement C1r and cathepsin S	LS-MS
[36] Yue X 2019	BALF	To explore the pathogenic mechanisms of ARDS due to direct and indirect pulmonary insult	control, intratracheal (I.T., direct) and intraperitoneal (I.P., indirect) LPS-treated mice	1017 proteins were identified; The two LPS groups shared 13 up-regulated and 22 down-regulated proteins compared to the control group	↑: Apcs, H2afz, Hba-a1, Hist2h2aa2 and Hmgn2 ↓: Anpep, Annexin A5, AU021092, Cadm1 and Cd200	Discovery-Based Quantitative Shotgun Proteomics

*BALF* Bronchoalveolar lavage fluid, *DIGE* Difference in-gel electrophoresis, *iTRAQ* Isobaric tags for relative and absolute quantitation, *MALDI-TOF/MS* Matrix-assisted laser desorption/ionization-time of flight/mass spectrometry, *SELDI* Surface-enhanced laser desorption ionization, *2D-HPLC* Two-dimensional high-performance liquid chromatography, *2D-PAGE* Two-dimensional polyacrylamide gel electrophoresis, *LC-MS* Liquid chromatograph-mass spectrometer, *ESI-MS* Electrospray ionization-mass spectrometer, *VAP* Ventilator associated pneumonia, *SFJDC* ShuFengJieDu Capsule, *ACB* Alveolar-capillary barrier, *↑* up-regulated proteins, ↓ down-regulated proteins, *GST* Glutathione S-transferase, *S100A8* calgranulin A, *S100A9* calgranulin B, *C3* Complement component C3, *α1-AGP1* Alpha-1-acid glycoprotein 1, *CRP* C-reactive protein, *SAA* serum amyloid A protein, *SERPINF1* serpin peptidase inhibitor, clade F, member 1, *GSTP1* glutathione S-transferase pi-1. *PEX13* Peroxisome biogenesis factor13, *HSP27* Heat shock protein 27, *MMP 9* matrix metallopeptidase 9, *GAPD* Glyceraldehyde-3-phosphate dehydrogenase, *TCTP* Translationally controlled tumor protein, *VDBG* Vitamin-D binding protein, *GSTA4* Glutathione S-transferase alpha-4, *LGALS5* Lectin, galactose binding, soluble 5, *SBP1* Selenium-binding protein 1, *C1qA* Complement C1q subcomponent subunit A, *ASCC3* Activating signal cointegrator 1 complex subunit 3, *GNAI1* Guanine nucleotide-binding protein G(i) subunit alpha-1, *VAMP3* Vesicle-associated membrane protein 3, *TNFAIP8* tumor necrosis factor α-induced protein 8, *MAPRC1* membrane-associated progesterone receptor component 1, *DNAJB11* DnaJ homolog subfamily B member 11, *PRKCDBP* Protein kinase C delta-binding protein, *DNAJC5* DnaJ homolog subfamily C member 5, *MAP6* microtubule-associated protein 6, *RASL11A* RAS-like family 11 member A, *LOC689092* Predicted: N-acetyllactosaminide β-1,6-N-acetylglucosaminyl-transferase, *NUDT5* Predicted: ADP-sugar pyrophosphatase isoform X1, *ENO1* Eno1 protein, *NEMF* Nuclear export mediator factor, *CHDH* Choline dehydrogenase, *Ankrd24* Ankyrin repeat domain 24, *TEC* TEC protein tyrosine kinase, *EPC1* Predicted: Enhancer of polycomb homolog 1 isoform X2, *ICAM-1* intercellular cell adhesion molecule-1, *VCAM-1* vascular cell adhesion molecule-1, *Apcs* Serum amyloid P-component, *H2afz* Histone H2A.Z, *Hba-a1* Hemoglobin subunit alpha, *Hist2h2aa2* Histone H2A type 2-A, *Hmgn2* Non-histone chromosomal protein HMG-17, *Anpep* Aminopeptidase N, *AU021092* UPF0764 protein C16orf89 homolog, *Cadm1* Cell adhesion molecule 1, *Cd200* OX-2 membrane glycoprotein

### Samples in non-targeted proteomics of ARDS

#### Bronchoalveolar lavage fluid (BALF) proteome in ARDS

BALF is one of the most common sources of samples for the study of lung diseases. The studies of pathophysiological mechanism of ARDS revealed that alveolar epithelial cells and pulmonary capillary endothelial cells are damaged, and the increased pulmonary vascular permeability leads to exudative pulmonary edema [24, 27]. These pathophysiological changes may be explained by changes in the protein profile of alveolar lavage fluid. In 2004, Bowler et al. [23] first applied proteomic approaches to ARDS research. They used two-dimension (2-DE) technology and matrix-assisted laser desorption/ionization time-of-flight mass spectrometry (MALDI-TOF-MS) technology and compared the BALF and plasma samples of ARDS patients with healthy people. This study found that some proteins were modified in many ways during lung injury, and these proteins could be identified by proteomic strategies at the time, but not by microarray, enzyme-linked immunosorbent assay (ELISA) or immunoblotting test or other identification methods. This method not only confirmed the existence of multiple subtypes of a single gene product in different disease states, but also demonstrated the potential and advantages of proteomic analysis in ARDS research.

With the development of proteomic technology, many low-abundance proteins that cannot be identified by traditional technologies have been identified, and together with known proteins, ARDS proteomic database has been constructed. Schnapp et al. [25] used a shotgun proteomic approach (2D-HPLC-MS/MS) to analyze BALF proteomic profiles from three ARDS patients and compared them with 6 healthy people. They showed that proteins of biological significance, such as insulin-like growth factor binding protein-3 (IGFBP-3), which were not previously identified in the BALF of ARDS patients, were followed by the ELISA method. It was verified that IGFBP-3 was significantly higher in the early stage of ARDS patients than in the normal control group. The author believed that IGFBP-3 down-regulated the expression of insulin-like growth factor in patients with ARDS and leaded to fibroblast apoptosis. At the same time, compared with the traditional proteomics methods, the identified proteins increased by nearly 10 times, suggesting that the shotgun technique is more comprehensive and reliable in identifying the ARDS protein profile.

Moreover, with the assistance of proteomics research, it is also possible to dynamically observe the changes of BALF protein during ARDS. Chang et al. [27] used 2DE-MALDI-TOF-MS technology to analyze BALF proteins of patients with ARDS on day 1, 3 and 7 after disease onset, and identified 37 proteins, most of which did not change significantly at three-time points, only a few proteins changed significantly, including annexin A3, surfactant protein A, actin, etc. The dynamic changes of BALF proteins not only reflect the repair of lung damage, but may also predict the prognosis of ARDS patients.

Furthermore, in another study [19], the authors divided ARDS patients into three groups: early survivors (1–7 days after the onset of ARDS), early non-survivors, and late survivors (8–35 days after the onset of ARDS), and to compare and analyze the BALF of three groups by isotope tags for relative and absolute quantification (iTRAQ). Not only the dynamic changes of lung protein expression in early and late ARDS were found, but also the difference in protein expression between ARDS survivors and non-survivors was found. These differential proteins reflect a coordinated compensation response to injury and stress in early survivors. Confirmed by ELISA, Clara cell secretory protein, Moesin, Matrix metalloproteinase 9 (MMP-9), Mucoprotein 5 AC and other proteins have been proved to be significantly different between the survival group and the non-survival group, which can be used as a potential biomarker to evaluate the prognosis of ARDS patients.

#### Serum or plasma samples proteome in ARDS

Compared with BALF, serum or plasma samples are relatively easy to obtain, and also contain some proteins associated with ARDS that may not be present in BALF, which is important to fully understand the pathophysiological mechanisms of ARDS. However, at the same time, high abundance proteins in serum may hinder the study of low abundance proteins [12, 35].

Chen et al. [37] combined the proteomic profile of inflammatory mediators together, using microarray technology to perform serum analysis on the normal control group, the bacterial-infected pneumonia group, and the pneumonia-ARDS group at three-time points (the day of the hospital, the third day, and the seventh day). Comparative analysis revealed 13 specific biomarkers for ARDS candidates. These candidate markers were evaluated through a digital evaluation scoring system, and the results were significantly related to clinical informatics. This research suggested that although microarray technology does not belong to the scope of proteomics technology, its large-scale study of protein characteristics can be a good method for studying proteomics.

In summary, limited and preliminary serum or plasma proteomic studies on ARDS may provide novel biomarker candidates and new insights into the pathogenesis of ARDS.

#### Lung tissues proteome in ARDS

The rat/mouse model of ARDS induced by various factors is often used to study the lung proteomics of ARDS [31]. Although the protein expression of lung tissue is inevitably different between mice and humans, these results can still provide a very important reference for the study of human ARDS. Also, some studies used 2-DE-based proteomic technology to study the characteristics of the lung tissue protein profiles obtained from patients with chronic obstructive lung disease (COPD) [13, 38]. However, as far as we know, there is still a lack of proteomic studies focusing on the identification and quantification of lung tissue proteins obtained from patients with ARDS. This is mainly due to the difficulty in obtaining sufficient lung tissue in critically ill patients. This is crucial because, compared with plasma proteomics, lung-specific proteomics is more capable of finding reliable and valuable biomarkers for the diagnosis, prognosis, pathogenesis and treatment of ARDS.

#### Lung-related cells proteome in ARDS

Alveolar macrophage (AM), the main defense cell in the airway, plays an important role in the pathogenesis and evolution of ARDS due to its role in phagocytosis and antigen presentation. When activated, they can secrete various cytokines or inflammatory factors to cause cascade inflammation [39, 40]. Dong et al. [30] believed that in addition to initiating, amplifying, and maintaining inflammatory response during the ARDS exudation period, AM also played a role in relieving persistent inflammation and preventing further tissue damage during the recovery period. Proteomic analysis and comparison of AM showed that the expression of 10 proteins significantly increased during the exudation period, and 17 proteins were significantly expressed during the recovery period, indicating that these 27 proteins were significantly related to the exudation period and the recovery period, respectively. Further analysis revealed that these proteins mainly played a role in regulating inflammation, oxidative stress, apoptosis and metabolism, and they had the potential to become biomarkers for early diagnosis and prognosis assessment of ARDS.

In another study, Bhargava et al. [41] studied the role of alveolar type II epithelial cells (AT II) in restoring the normal structure of alveoli in the injured lung and used proteomics methods to test AT II cells during the injury and recovery period of hyperoxia-induced ALI rat model. It was found that 183 kinds of proteins changed significantly from the injury to recovery period. Based on these data, the author also established a new algorithm to identify the protein clusters that change during the damage and repair of AT II cells, which provided an important basis for further research on the molecular mechanism of lung injury repair.

#### Exhaled breath proteome in ARDS

Exhaled gas condensates (EBC) contain small amounts of proteins that leave the lungs through the production of aerosol droplets [26, 42]. EBC’s protein model may be useful for monitoring acute and severe lung diseases, mainly for monitoring inflammatory lung diseases, such as asthma, COPD, interstitial lung disease and ARDS, especially monitoring the pressure during mechanical ventilation. Gessner et al. [26] demonstrated an increased frequency of cytokeratin detection in EBC samples from mechanically ventilated patients with ARDS. The increase of cytokeratin detection rate was associated with higher PIP and PEEP levels, more severe lung injury and longer ventilation time. Therefore, the identification of markers of tissue injury indicating mechanical response during mechanical ventilation may provide an opportunity for future ventilation patterns to respond to lung tissue.

### Study designs in non-targeted proteomics of ARDS

Concerning ***the direct (pulmonary) or indirect (extra-pulmonary) insults***. In pulmonary ARDS, direct injury mainly affects alveolar epithelium with local alveolar inflammatory reaction, while in extrapulmonary ARDS, indirect injury affects vascular endothelium through inflammatory mediators in blood flow, showing more serious endothelial damage [36]. The pathogenesis of ARDS caused by direct and indirect lung injury is not fully understood. In a study [28], 26 patients with ARDS were divided into two groups: direct lung injury and indirect lung injury based on the etiology. For the first time, iTRAQ and MALDI-TOF-MS were used to perform proteomic analysis on the serum of each group of patients, and a total of 16 identified protein expression differences (compared with the normal control group) were found, of which 11 proteins were identified in both groups, while the other 5 proteins were only identified in the direct lung injury group. Through bioinformatics analysis, it was found that these differential proteins were mainly involved in lipid metabolism/transportation, immune system processes and other biological processes, and the acute phase response was the most important signal pathway.

Concerning ***the ARDS model mediated by various factors***. Liu et al. [31] analyzed the proteome of lung tissue of ALI rats induced by ***Pseudomonas aeruginosa*** for the first time, and found that the expression levels of 18 kinds of proteins changed twice or more compared with the normal control group, which mainly involved in biological processes such as energy metabolism, antioxidant, protein binding and signal transduction. Among them, human antioxidant protein-1 (PRDXl) is considered to be the promoter of the inflammatory mechanism of ARDS. Western blot was used to confirm that PRDXl played a key role in promoting the inflammatory response of ARDS. Xu et al. [33] carried out a proteomic analysis on lung tissue of ALI mice induced by ***oleic acid*** and ***saline***-treated mice by iTRAQ technology. After verification by Western blot, proteins such as antithrombin III (AT III), 12-lipoxygenase and cytokine-2 detoxification were selected as candidate biomarkers of ALI mice induced by oleic acid. After that, siRNA interference was used to study the effect of AT III on the integrity of pulmonary endothelial cells. The results showed that the expression level of inflammatory factors increased and the increased permeability of endothelial cells after AT III gene was deleted, indicating that AT III played an important role in oleic acid-induced ALI mice. In another study, Sakaue et al. [43] established a mouse model of ALI secondary to the ***liver injury induced by ligation of common bile duct***, and carried out proteomic analysis on lung tissue. The results showed that compared with the control group, the expression of serine protease inhibitor Bla (serpin Bla), Annexin A1 (anxal) and calcium-binding protein A9 (S100A9) were significantly increased in the experimental group. Subsequently, immunohistochemistry technology and quantitative Reverse Transcription-Polymerase Chain Reaction (RT-PCR) technology were used to study lung tissue and liver tissue of mouse. The results of immunohistochemistry showed that the above three proteins were highly expressed in the pulmonary blood vessels in the ALI mouse model, and the quantitative RT-PCR results showed that serpin Bla was up-regulated in the liver, and S100A9 and anxal were up-regulated in the lung. This study linked liver injury with lung injury, and identified three proteins that may be involved in the pathological process of liver injury leading to lung injury, providing new insights into the diagnosis and treatment of ARDS. Xu et al. [44] confirmed that HA330 resin-directed blood adsorption could reduce pulmonary edema and inflammatory damage caused by ***Lipopolysaccharide (LPS)*** by removing inflammatory factors in ARDS pig model proteomic study. Besides, some studies [45] reported that hydrogen has a protective effect on sepsis-related ALI. In this study, 192 differentially expressed proteins were found to be related to the mechanism of hydrogen action in sepsis-related ALI mice model induced by ***cecal ligation and puncture***. These results provide new insights into the prevention or treatment of sepsis-related ALI by hydrogen.

Concerning ***the different periods of ARDS***. Some studies [30] took the alveolar macrophages of patients with sepsis ARDS as the research object, using proteomics methods to study and analyze them in the early course of ARDS (within 24 h of onset) and on the 5th day of onset (significant improvement). It was found that 17 proteins were significantly increased during the recovery phase, while the remaining 10 proteins were up-regulated in the early stages of the disease. The above-mentioned proteins play a role in regulating inflammation, cytoskeleton organization, oxidative stress, apoptosis and metabolism. They may be used as biomarkers for the early diagnosis and prognosis of ARDS patients [27].

Concerning ***the complications of ARDS***. Ventilator-associated pneumonia (VAP) is a common complication in patients with ARDS. Nguyen and colleagues [29] obtained BALF from 5 normal subjects and 30 patients with ARDS which included 14 patients with VAP (VAP (+)), and 16 patients without (VAP (−)).In the ARDS group, they identified 76 differentially expressed proteins between HAP(+) and VAP(−). The functional analysis of these proteins indicated that the pro-inflammatory pathway was activated during VAP. They identified and verified a limited proteomic feature that can distinguish VAP(+) from VAP(−) patients, which is composed of the following three proteins: S100A8, Lactoferrin (LTF) and Actin 1 (ACTN1).

Concerning ***severe acute respiratory syndrome coronavirus 2(SARS-CoV-2)-infected host cells***. As ARDS caused by the new coronavirus SARS-CoV-2 is raging around the world, coronavirus disease 2019 (COVID-19) is highly contagious [46, 47]. With the help of unbiased proteomic technology, the infected cells can be detected to reveal the biological pathways and potential drug targets related to virus pathogenesis. However, this technology relies on the cell model of virus transient infection and the related high sensitivity proteomic methods. In the recent study [48], the author’s team successfully isolated the SARS -cov-2 virus from the human colon epithelial carcinoma cell line Caco-2, and established the cell model. On the omics method, the team has recently developed a proteomic method called mePROD (multiplexed enhanced protein dynamics), which is used to deal with protein samples with weak label signals due to short-term processing. This method is based on stable isotope labeling by amino acids in cell culture (SILAC) labeling technology, which will not affect the cell itself, so it can be used to analyze the virus infected cells without deviation and interference. In this paper, the author used mepro D technology to detect proteins at different time points after the virus infection, determined the biological process related to infection, and then carried out the detection of potential drugs. These drugs could inhibit the replication of SARS -cov-2 at the concentration of non-toxic to human cells, which might provide therapeutic strategies for the treatment of COVID − 19.

### Limitations in current studies

As mentioned above, up to now, the research of non-targeted proteomics is limited by many internal and external factors. For different biological pathways, ARDS is a syndrome caused by different pathophysiological disorders, which limits the value of a single biomarker specific to a biological pathway. Models of multiple biomarkers from different biological pathways may be needed to establish reliable biological standards for ARDS [8]. Also, intriguingly, non-targeting techniques in proteomics are becoming an important way to find biomarkers related to ARDS.

For proteomic technology, some proteome, including low abundance proteins, membrane and hydrophobic proteins, as well as proteins with high molecular weight or extremely low or high pH, cannot be well separated by 2-DE and therefore cannot be detected by subsequent mass spectrometry [49]. Although the coupling of LC and MS significantly improves the separation, identification and quantification of small or hydrophobic proteins, it is still impossible to detect proteins of relatively low levels in various biological mixtures, such as chemokines, cytokines, growth factors, intracellular signaling proteins or transcription factors [50].

For the collection time of samples, the early collection of reliable timing sample biobank in the development of ARDS may enhance the efforts to deduce the biological signals of ARDS;

For sample selection and sample size, ARDS has wide heterogeneity, and small sample size may lead to heterogeneous conclusions. Currently, most studies choose plasma, but plasma and lung tissue are quite different; However, BALF is not easy to obtain. Some studies have shown that edema fluid can be directly sampled in the early stage of ARDS [11]. What’s more, with the improvement of proteomic assessment of exhalation [12], this may be an attractive option to enhance the temporal/longitudinal evaluation of future studies.

Furthermore, most studies have not classified etiology. Calfee’s study [51] used large clinical samples to classify the subtypes of ARDS, including a simplified model of two biomarkers (IL-6 and soluble TNFR-1) and clinical variables of vasopressor use at baseline, which correctly classified phenotypes in both populations with an accuracy of > 90%. This suggests that this phenotype may be evaluated as a target population in future clinical trials.

## Conclusions

### Future preview and application of ARDS therapy

At present, there is no specific drug treatment for ARDS. Neuromuscular blocker is only an auxiliary drug of lung protection ventilation strategy. Proteomic methods can not only study the whole set of proteins of ARDS, find out the key target as the cut-off point of drug treatment, but also verify the drugs that may be effective treatments for ARDS, and study the possible mechanism of their intervention in the treatment of ARDS [8, 52]. Some studies [53] carried out a proteomic analysis on lung tissue of rats with ALI induced by high tidal volume ventilation, and found that the activity of matrix metalloproteinase-9 in lung was decreased after doxycycline treatment. Therefore, the authors suggested that doxycycline may prevent or treat high tidal volume ventilation-induced ALI by inhibiting the activity of matrix metalloproteinase-9. Certain Chinese medicine preparations such as ShuFengJieDu Capsule [34] and Jie-Geng-Tang [32] have certain therapeutic effects on ARDS, but the mechanism is unknown. Proteomic research can explain the possible potential effects of drugs.

In summary, researchers use proteomics technology to study samples of BALF, serum or plasma, lung tissue, lung cells and others from ARDS patients or animal models, revealing the pathophysiological mechanism of ARDS, screening new candidate biomarkers, searching for therapeutic targets and developing new drugs. At present, although the application of proteomic technology in the pathogenesis of ARDS is just started, its great potential has been shown in deepening the understanding of protein expression patterns in ARDS, discovering new damage mediators and developing new therapeutic drugs. After the completion of the human genome project, human proteome has become the main human science project [54], and the establishment of a proteome database of a single disease will also be the trend of disease research in the future [13].

## Data Availability

Not applicable.

## Abbreviations

ARDS: Acute respiratory distress syndrome
ALI: Acute lung injury
ICU: Intensive care unit
BALF: Bronchoalveolar lavage fluid
DIGE: Difference in-gel electrophoresis
iTRAQ: Isobaric tags for relative and absolute quantitation
MALDI-TOF/MS: Matrix-assisted laser desorption/ionization-time of flight/mass spectrometry
SELDI: Surface-enhanced laser desorption ionization
2D-HPLC: Two-dimensional high-performance liquid chromatography
2D-PAGE: Two-dimensional polyacrylamide gel electrophoresis
LC-MS: Liquid chromatograph-mass spectrometer
ESI-MS: Electrospray ionization-mass spectrometer
VAP: Ventilator associated pneumonia
SFJDC: ShuFengJieDu Capsule
ACB: Alveolar-capillary barrier
ELISA: Enzyme-linked immunosorbent assay
IGFBP-3: Insulin-like growth factor binding protein-3
IGF: Insulin-like growth factor
MMP-9: Matrix metalloproteinase 9
AM: Alveolar macrophage
AT II: Alveolar type II epithelial cells
EBC: Exhaled gas condensates
PRDX1: Human antioxidant protein-1
AT III: Antithrombin III
PT-PCR: Reverse Transcription-Polymerase Chain Reaction
LPS: Lipopolysaccharide
SARS-CoV-2: Severe acute respiratory syndrome coronavirus 2
COVID-19: Coronavirus disease 2019
